# The Principles and Practice of Medicine

**Published:** 1892-09

**Authors:** 


### Reviews of Books.

*The Principles and Practice of Medicine.* By WILLIAM OSLER, M.D., F.R.C.P. Pp. xvi., 1079. Edinburgh: YOUNG J. PENTLAND. 1892.

It is difficult to compile a text-book of medicine and to bring it within reasonable limits. The author of this one plunges boldly into typhoid fever, and completes the book in a little over one thousand closely printed pages, in which padding is nowhere to be found, but each subject is dealt with from the original standpoint by an experienced physician, who is thoroughly conversant with the questions on which he writes, and who describes briefly what is fairly well established knowledge without branching off into side issues or theoretical discussions.

The selection of any particular topic for comment is not easy, inasmuch as the author gives little scope for criticism. In the treatment of gastric ulcer, we read that "for the vomiting there is no measure so successful as lavage," and that "ill effects rarely follow the careful use of the stomach-tube." We should not run the risk of ill effects by using the tube, but would prefer to adopt peptonised rectal feeding if the vomiting continued after everything but small doses of peptonised milk had been withheld. As regards the occurrence of hemorrhage, we are glad to find that the author has the courage to advise that "no attempt should be made to check the hemorrhage by administering medicines through the mouth; as the profuse bleeding is always from an eroded artery, frequently from one of considerable size, it is doubtful if . . . the usual remedies have the slightest influence. The essential point is to give rest, which is best obtained by opium. . . . Nothing should be given by the mouth except small quantities of ice."

In dealing with the treatment of phthisis, we are sorry to find that so experienced and trustworthy an observer should come to the conclusion that "no medicinal agents have any special or peculiar action upon tuberculous processes." Creasote and guaiacol do not appear to have been given in doses exceeding ten minims, a quantity which scarcely justifies the conclusion that "the remedy has no essential influence on the progress of the disease." The author insists on the examination of sputum in the early diagnosis of phthisis, and remarks: "There is no excuse now for its omission, since, if the practitioner has not command of the necessary *technique*, there are laboratories in many parts of the country at which the examination can be made." We have no doubt the author would join with us in a protest against the principle of entrusting the examination of secretions to non-medical analysts: such specialisation as this is by no means to be encouraged; surely a medical practitioner, however busy he may be, can find time to make the necessary investigations, and would hesitate to draw any inference from observations which he had not himself confirmed. The time has not yet come when our patients should bring with them a report on the condition of the various secretions, and when the physician himself should be absolved from the duty of making his own investigation, whether chemical, microscopic, or physical.

We must refrain from further diving into a book which cannot fail to be a good friend to those who need a new text-book of medicine. It is well printed in easy type; it has a full index and not many mistakes, but "ataxic" scarlet fever (page 71 and the index) is surely a printer's error.

We think, too, that no article on typhoid fever can be considered complete without mention of the name of Dr. William Budd, of Clifton, whose work established the specific nature of the disease and the contagious character of the intestinal discharges, even although he was not able to demonstrate the specific germ.

